# Prescribing practices of inhaled corticosteroids for premature infants in the neonatal intensive care unit

**DOI:** 10.1038/s41372-024-01891-w

**Published:** 2024-01-31

**Authors:** Monica Tang, Anna Ibrahim, Christopher Laughon, Kaila Moore, Angibel Tejada, Dean Tran, Ryan Kilpatrick, Rachel G. Greenberg, Christoph P. Hornik, Kanecia Zimmerman, Matthew M. Laughon, Reese H. Clark, Jason E. Lang

**Affiliations:** 1 Department of Medicine, University of California San Francisco, San Francisco, CA USA; 2 Duke Clinical Research Institute, Durham, NC USA; 3 Department of Pediatrics, Duke University, Durham, NC USA; 4 Department of Pediatrics, The University of North Carolina at Chapel Hill, Chapel Hill, NC USA; 5 Pediatrix Center for Research and Education, Pediatrix Medical Group, Inc, Sunrise, FL USA

**Keywords:** Drug therapy, Respiratory tract diseases

## Abstract

**Objective:**

Despite limited safety and efficacy data, inhaled corticosteroids (ICS) are prescribed to premature infants in the neonatal intensive care unit (NICU). We examined contemporary use and risk factors for ICS use in the NICU.

**Study design:**

Infants <33 weeks gestational age and <1500 gm birth weight discharged from Pediatrix Medical Group NICUs between 2010 and 2020 were included. We evaluated the association between ICS prescription and clinical characteristics using univariable and multivariable logistic regression.

**Results:**

Of 74,123 infants from 308 NICUs, 9253 (12.5%) were prescribed ICS: budesonide, fluticasone, or beclomethasone. Diagnosis of bronchopulmonary dysplasia (BPD), earlier gestational age, male sex, longer mechanical ventilation, oxygen support, and systemic steroids were independent risk factors for ICS prescription.

**Conclusions:**

Use of ICS is common in many NICUs and is associated with a diagnosis of BPD and healthcare utilization. Prospective trials are needed to establish the safety, efficacy, and optimal indication in this vulnerable population.

## Introduction

Inhaled corticosteroids (ICS) have been commonly prescribed to premature infants in the neonatal intensive care unit (NICU) [1–5]. However, ICS are not currently approved by The Food and Drug Administration (FDA) for use in children <12 months of age and are only currently approved for the maintenance treatment of asthma. The benefits of ICS in asthma may be limited to those with eosinophilic inflammation compared to those with other types of inflammation [6–8].

Previous studies of ICS use in the neonatal population have focused on the use of ICS in bronchopulmonary dysplasia (BPD) [1–5]. BPD is characterized by impaired lung development and is one of the leading causes of prematurity-related morbidity and mortality [9]. With the introduction of antenatal steroids and intratracheal surfactant, the morbidity and mortality rate of neonates with BPD is improving; however, there are still no U.S. FDA-approved drug therapies for infants to prevent or treat BPD [9, 10]. Studies supporting the use of ICS for BPD have been equivocal, and the long-term effects in premature infants are unknown (Supplemental Table 1) [11–18]. Currently there is insufficient evidence for expert organizations to recommend the routine use of ICS to prevent BPD or to treat the chronic respiratory symptoms of BPD [19, 20]. The objective of this study was to describe the contemporary use of ICS in NICUs across the US and to describe the risk factors for increased use.

## Methods

This was a cohort study of premature infants discharged between 2010 and 2020 from a NICU in the Pediatrix Medical Group, Inc. The Pediatrix Medical Group, Inc prospectively captures information on infants cared for in NICUs from 35 states and Puerto Rico [21]. Inclusion and exclusion criteria were chosen to identify infants with complete hospitalizations and associated medication exposure histories. This study with deidentified data was reviewed and determined to be exempt from IRB review by the Duke University IRB (Pro0092987).

### Subjects

We included infants with gestational age <33 weeks, birth weight <1500 g, and discharged after 14 days of age. Infants were excluded if they were <22 weeks gestation, not born in the hospital, transferred to another hospital, died during their hospitalization, or had a postmenstrual age (PMA) at discharge <33 weeks or >63 weeks to eliminate outliers.

### Definitions

Demographic variables of interest included gestational age, birth weight, sex, race/ethnicity, and postmenstrual age at discharge. Clinical variables of interest included delivery mode, respiratory diagnoses, respiratory support, and medications of interest. Respiratory diagnoses of interest included physician diagnosed persistent pulmonary hypertension in the neonate (PPHN), pneumonia, aspiration, reactive airway disease, asthma, bronchiolitis, wheezing, tracheomalacia, bronchomalacia, chronic pulmonary insufficiency, BPD, and chronic lung disease. We also used a study definition for BPD, which was requiring supplemental oxygen, nasal cannula, continuous positive airway pressure, conventional mechanical ventilation, or high-frequency ventilation in infants from a PMA of 36 0/7 to 36 6/7 weeks [22]. Medications of interest included exposure to antenatal steroids, use of surfactant, montelukast, systemic steroids (including dexamethasone, hydrocortisone, prednisolone, and prednisone), diuretics (including acetazolamide, bumetanide, chlorothiazide, diazoxide, ethacrynic acid, furosemide, hydrochlorothiazide, metolazone, prazosin, and spironolactone), sildenafil, and bronchodilators (including albuterol, ipratropium). Respiratory support included use of mechanical ventilation and supplemental oxygen defined as fraction of inspired oxygen  > 21% during their hospitalization. Individual NICU characteristics included annual total and BPD discharges and median gestational age.

### Statistical analysis

We used standard summary statistics, including medians (25th–75th percentiles) and counts (percentages) to describe continuous and categorical study variables, including duration of ICS prescription. We also determined the prevalence of ICS prescription by specific drug over time. The primary outcome variable (ICS prescription ever) was analyzed using univariable and multivariable logistic regression. We included presence of the study definition of BPD, gestational age, antenatal steroid use, duration (by week) of oxygen support, mechanical ventilation, and postnatal systemic steroid, race/ethnicity, and sex as fixed effects in the multivariable model. Site was included as a random intercept. The strengths of associations were estimated using odds ratios (ORs) and 95% confidence intervals (CIs). Variables were included in the multivariable logistic regression with backwards selection using variables with *p* < 0.2 identified by univariable analysis.

## Results

**Fig. 1:**
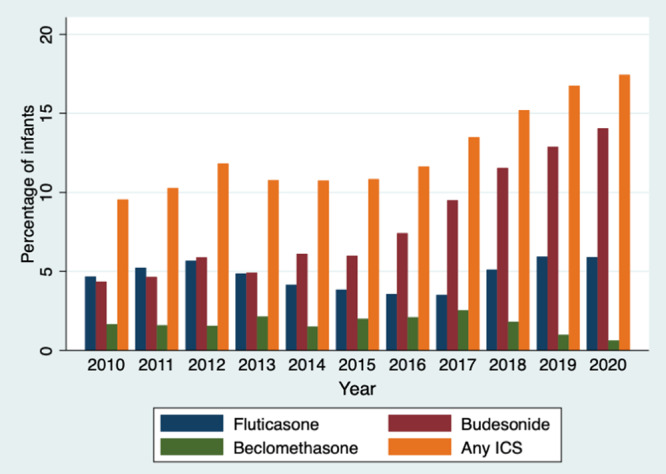
Inhaled corticosteroid prescription by year. ICS inhaled corticosteroid.

A total of 74,123 infants at 308 sites were included in the analyses, with 9253 (12.5%) infants prescribed ICS. Over the 11-year study period, annual ICS prescription has increased (Fig. 1). Budesonide was the most prescribed ICS overall and annually since 2012 (*n* = 5775, 7.8%), followed by fluticasone (*n* = 3529, 4.8%), and beclomethasone (*n* = 1255, 1.7%). There was considerable variation in ICS prescription by site. Among sites with at least 20 infants, the percentage of infants receiving ICS at each site ranged from 0 to 12% and 148 (65.8%) of sites did not prescribe ICS to any infants. Prescription of ICS in the NICU started at a median of 35 days (25th–75th percentile 21–56) at a median PMA 31 weeks (25th–75th percentile 29–34), ended at a median 69 days (25th–75th percentile 47–92) at a median PMA 36 weeks (25th–75th percentile 33–39), and for a median duration of 21 days (25th–75th percentile 5–44). ICS-prescribed infants were more likely to be male, have lower birthweight and gestational age than infants not prescribed ICS (Table 1). A study-defined diagnosis of BPD was made in 69% (*n* = 6358) of those prescribed ICS. Infants prescribed ICS also had a greater proportion diagnosed with other chronic respiratory conditions than infants not prescribed ICS. There were 584 (6%) infants prescribed ICS who did not have any of the respiratory diagnoses of interest documented. Infants prescribed ICS had a greater proportion receiving oxygen support and mechanical ventilation and for a longer duration than infants not prescribed ICS. Infants prescribed ICS were more likely to receive surfactant, diuretics, systemic steroids, and bronchodilators than infants not prescribed ICS. Additionally, infants prescribed ICS were born at NICUs with higher annual total and BPD discharges than infants not prescribed ICS.

**Table 1:** Characteristics of infants by inhaled corticosteroid prescription status.

	ICS (*n* = 9253)	No ICS (*n* = 64,870)
Cesarean section	7003 (77)	49,448 (77)
Birthweight (grams)		
≤500	464 (5)	495 (1)
501–1000	6582 (71)	19,826 (31)
1001–1500	2207 (24)	44,549 (69)
Gestational age (weeks)		
<25	3939 (43)	6117 (9)
26–28	4058 (44)	22,237 (34)
29–32	1256 (14)	36,516 (56)
PMA at discharge (weeks)	40 (38–43)	37 (35–38)
Male	5070 (55)	31,241 (48)
Race/ethnicity		
White	3687 (42)	26,846 (44)
Black	2939 (33)	18,734 (30)
Hispanic	1564 (18)	11,786 (19)
Other	583 (7)	4276 (7)
Chronic respiratory diseases		
Study defined BPD	6358 (69)	12,608 (20)
Provider defined		
Pulmonary insufficiency	4740 (51)	22,985 (35)
BPD	2221 (24)	3901 (6)
CLD	4559 (49)	9254 (14)
PPHN	1024 (11)	1577 (2)
Pneumonia	1294 (14)	2017 (3)
Aspiration	331 (4)	614 (1)
RAD/Asthma	36 (0.4)	38 (0.1)
Bronchiolitis/Wheezing	44 (0.5)	71 (0.1)
Tracheobronchomalacia	56 (0.6)	72 (0.1)
Oxygen support	9101 (98)	49,476 (76)
Duration in weeks	10 (5–15)	1 (0–4)
Mechanical ventilation	7971 (86)	29,825 (46)
Duration in weeks	3 (0–6)	0 (0–0)
Other medication use		
Antenatal steroid	8272 (89)	57,586 (89)
Surfactant	7557 (82)	34,689 (53)
Montelukast	7 (0.1)	3 (0.0)
Diuretic	6935 (75)	15,744 (24)
Bronchodilator	4578 (49)	2657 (4)
Postnatal systemic steroid	4572 (49)	6289 (10)

Values reported as median (25th–75th percentile) for continuous variables and counts (percentages) for categorical variables.

*ICS* inhaled corticosteroid, *PMA* postmenstrual age, *BPD* bronchopulmonary dysplasia, *CLD* chronic lung disease, *PPHN* persistent pulmonary hypertension of the newborn, *RAD* reactive airway disease.

In univariate logistic regression analyses, factors associated with an increase in the odds of ICS prescription included male sex, Black race, earlier gestational age, BPD diagnosis, and longer duration of supplemental oxygen, mechanical ventilation, and postnatal systemic steroids (Table 2). Multivariate logistic regression identified male sex, earlier gestational age, BPD diagnosis, duration of oxygen support, mechanical ventilation, and postnatal systemic steroid use as independent risk factors for ICS prescription.

**Table 2:** Predictors of inhaled corticosteroid prescription in the neonatal intensive care unit.

	Unadjusted OR (95% CI)	Adjusted OR (95% CI)
Risk factors:		
Gestational age		
<25 weeks	**18.72 (17.47–20.06)**	**1.66 (1.48–1.87)**
26–28 weeks	**5.31 (4.97–5.66)**	**1.69 (1.54–1.85)**
29–32 weeks	ref	Ref
Male sex	**1.30 (1.25–1.36)**	**1.16 (1.09–1.24)**
Race/ethnicity		
White	ref	ref
Black	**1.14 (1.08–1.20)**	0.99 (0.91–1.08)
Hispanic	0.97 (0.91–1.03)	0.93 (0.84–1.03)
Other	0.99 (0.90–1.09)	**0.86 (0.75–0.99)**
BPD	**9.23 (8.79–9.69)**	**2.50 (2.27–2.75)**
Antenatal steroid use	1.07 (0.99–1.14)	**1.29 (1.16–1.44)**
Mechanical ventilation (weeks)	**1.47 (1.46–1.48)**	**1.02 (1.00–1.04)**
Oxygen support (weeks)	**1.24 (1.23–1.24)**	**1.22 (1.20–1.23)**
Systemic steroid use (weeks)	**1.41 (1.39–1.43)**	**1.08 (1.06–1.10)**

Values reported as odds ratio (95% confidence interval).

*PMA* postmenstrual age, *BPD* study defined bronchopulmonary dysplasia. Analysis performed by simple and multivariable logistic regression with backwards selection using variables with *p* < 0.2 identified by univariable analysis. Bold indicates statistical significance.

## Discussion

This study of premature infants born <33 weeks showed that ICS is prescribed off-label with regularity at many NICUs in the US. Diagnosis of BPD, male sex, gestational age, and healthcare utilization predicted ICS prescription. There was variability in ICS prescription by site, but the annual number of total and BPD discharges was associated with an increased odds of ICS prescription. We saw no evidence of a decline in use over the decade under study of this off-label therapy, suggesting the high need for improve therapies to prevent and treat BPD.

Previous analyses were limited to surveys of providers or populations of infants with BPD (Supplemental Table 1) [1–5]. We examined ICS use independent of diagnosis as the Pediatrix dataset captures prescription medications and diagnoses separately. Most infants prescribed ICS met the standardized criteria for BPD diagnosis, and the most common provider diagnosed conditions were chronic pulmonary insufficiency, chronic lung disease, and BPD. Due to the retrospective nature of our study, it is possible that some infants had BPD not captured by our definition, incomplete documentation, or another physician determined indication. This study corroborated real-world ICS use in infants <33 weeks predominantly in those either diagnosed or meeting criteria for BPD.

Oxygen support, mechanical ventilation, and postnatal systemic steroids were associated with ICS prescription. Previous studies have shown mixed efficacy of ICS for short-term benefits including earlier extubation, reduced supplemental oxygen need, and less systemic steroid use (Supplemental Table 1) [11–18, 23]. However, long term safety is not well established and the largest follow-up study found infants given inhaled budesonide had a higher mortality rate at 2 years than those given placebo [12]. Additionally, systematic reviews are challenging given the significant heterogeneity in the studies for the use of ICS for BPD. The studies were designed for different indications (prevention vs. treatment), patient populations, drugs (beclomethasone, budesonide, or fluticasone) at different doses, administration timing, and durations. The variability in ICS prescription is likely related to the lack of FDA guidance on prescription of ICS, and the limited options for neonatologists for treating severe respiratory failure and BPD.

We were unable to assess ICS use after discharge, but a longitudinal analysis of 130 children with severe BPD found sustained use of ICS in 35–40% from NICU discharge to 2 years of age [24]. The Bronchopulmonary Dysplasia Collaborative, a consortium of 12 US institutions studying BPD, currently recommends treating reactive small airway disease similarly to childhood asthma with ICS [25]. In their review, the expert group also discusses the paucity of data in these patients and recommends further evaluation to determine the appropriate dose, time, and type of steroid.

Current off-label use of ICS in the NICU is substantial and associated with a diagnosis of BPD, duration of respiratory support, and medication utilization. Using drugs off label and without high quality studies exposes infants to drugs that might not work and may be harmful. Future research is needed to determine not only the efficacy and safety of ICS for prevention of BPD, treatment of BPD, and treatment of asthma symptoms in children with a history of BPD, but also the dosing, timing, and type of ICS best for these at-risk populations.

### Supplementary information

Supplemental Table 1

## Data Availability

The datasets generated and analysed during the current study are available from the corresponding author on reasonable request.
