# Supplementary material for: Prescribing practices of inhaled corticosteroids for premature infants in the neonatal intensive care unit
Source: J Perinatol. 2024 Jan 31;44(7):953–6. doi: 10.1038/s41372-024-01891-w (PMC11226392; doi:10.1038/s41372-024-01891-w)
Supplement: Supplementary file 1 — Supplemental Table 1 [file 41372_2024_1891_MOESM1_ESM.docx]

**Supplemental Table 1: Summary of previous studies on inhaled corticosteroids in premature infants.**

| Use of inhaled corticosteroids in premature infants | | |
| --- | --- | --- |
| Study | Methods | Results |
| Williams and Greenough 2003^1^ | Survey of UK neonatal units | - 40% (64/160) prescribed inhaled and systemic corticosteroids. 4% (6/160) prescribed inhaled corticosteroids only. - ICS were used for established CLD and/or wheeze. - ICS were not prescribed in the first 2 weeks after birth. |
| Porter and Gerner 2005^2^ | Survey of US institutions with neonatal fellowship programs in 2003 | - For prevention of BPD, 10% (5/51) used ICS alone and 14% (7/51) used ICS or systemic corticosteroids. - For treatment of BPD, 10% (5/51) used ICS alone and 69% (35/51) used ICS or systemic corticosteroids. |
| Maas et al. 2010^3^ | Survey of German hospital neonatal units in 2009 | - 46% (102/223) used ICS to care for preterm infants. - Main reasons given for ICS were lack of improvement despite other treatment strategies, prolonged duration of oxygen or mechanical ventilation. |
| Slaughter et al. 2014^4^ | Retrospective cohort study of infants born at <29 weeks gestation, birth weight <1500g from the PHIS database from 2007-2011 | - 25% (352/1429) received ICS. - Gestational age, birth weight, and prolonged ventilation all increased odds of receiving ICS. - Wide variations between institutions. |
| Yao et al. 2022^5^ | Retrospective cohort study of infants born <32 weeks gestation and admitted to a NHS neonatal unit between 2012-2019 | - 1% (922/62019) received inhaled budesonide. - Inhaled budesonide use increased from 2012-2019. - Inhaled budesonide was started at median postnatal age 42 days (IQR 30-70). |
| Efficacy of inhaled corticosteroids in premature infants | | |
| Study | Methods | Primary results |
| Bassler et al. 2015 and 2018^11,12^ | RCT of inhaled budesonide vs placebo within 24 hours of life in extremely preterm infants requiring positive pressure respiratory support between 2010-2013 | - N=437/863 received inhaled budesonide - Significant reduction in BPD at 36 weeks PMA - Increased mortality rate at 2 years. - No significant difference in the rate of neurodevelopmental disability at 2 years. |
| Clouse et al. 2016^13^ | Systematic review of inhaled corticosteroids in infants with BPD | - N=2383 infants from 14 studies. - Unable to do meta-analysis due to the study heterogeneity and quality. - Mixed results regarding efficacy. |
| Shinwell et al. 2016^14^ | Meta-analysis of ICS for prevention or treatment of BPD or death in preterm infants | - N=1596 infants from 16 studies - Significant reduction in BPD at 36 weeks PMA - No significant effect on mortality rate at 28 days or 36 weeks. |
| Shah et al. 2017^15^ | Systematic review of ICS within first two weeks of life for prevention of BPD in VLBW preterm infants | - n=1644 infants from 10 trials. - Significant reduction in the incidence of death or chronic lung disease at 36 weeks PMA |
| Shah et al. 2017^17^ | Systematic review of ICS vs systemic corticosteroids within first 7 days of life for prevention of BPD in VLBW preterm infants | - n=294 infants from 2 trials. - No significant difference in the incidence of death or chronic lung disease at 36 weeks PMA. - No evidence that ICS have advantages over systemic corticosteroids. |
| Shah et al. 2017^16^ | Systematic review of ICS vs systemic corticosteroids after the first week of life for treatment of BPD in VLBW preterm infants | - n=431 infants from 3 trials. - No significant difference in the incidence of death or chronic lung disease at 36 weeks PMA. - No evidence that ICS have advantages over systemic corticosteroids. |
| Onland et al. 2022^18^ | Systematic review of ICS between 1-36weeks PMA to preterm infants | - N=218 neonates from 7 trials - Low certainty evidence with no significant association between ICS and mortality or BPD at 36 weeks PMA |
